# Study partners’ views on remuneration in longitudinal Alzheimer’s disease research

**DOI:** 10.1002/bsa3.70048

**Published:** 2025-11-06

**Authors:** Erin D. Solomon, Matthew Gabel, Semere Bekena, Spondita Goswami, Krista L. Moulder, John C. Morris, Jessica Mozersky

**Affiliations:** 1 Bioethics Research Center, Washington University, St. Louis, Missouri, USA; 2 Department of Political Science, Washington University, St. Louis, Missouri, USA; 3 Knight Alzheimer Disease Research Center, Washington University, St. Louis, Missouri, USA

**Keywords:** Alzheimer’s disease, dementia, health disparities, racial and ethnic minorities, recruitment, research participation, retention, underrepresented groups

## Abstract

**Introduction:**

Recruiting and retaining participants in Alzheimer’s disease (AD) research poses a consistent challenge. Remuneration is one “readily modifiable factor” that could improve recruitment and retention in longitudinal observational AD research, and guidance suggests that all participants and their study partners receive remuneration for participating in AD longitudinal research.

**Methods:**

We surveyed study partners (*N* = 517) at one Alzheimer’s Disease Research Center in the United States regarding their views on remuneration.

**Results:**

Study partners felt largely neutral to positive regarding remuneration. Self-identified Black study partners had more positive views on remuneration than White study partners. Study partners with the least favorable views on remuneration perceived a lower participation burden and were less motivated by the perceived personal benefits of participating. All study partners were motivated by altruism and committed to continued participation.

**Discussion:**

Most study partners perceive remuneration as acceptable. Remuneration may improve recruitment and retention of historically underrepresented groups.

## 1 | INTRODUCTION

The National Institute on Aging (NIA) supports 36 Alzheimer’s Disease Research Centers (ADRCs) throughout the United States.^1^ Each of those centers is required to maintain a longitudinal study cohort focused on investigating the natural progression of Alzheimer’s disease (AD). These cohorts are observational longitudinal cohorts. Each participant in the cohort is required to have a study partner, also called a collateral source, who must also enroll in the longitudinal cohort alongside the participant. The role of a study partner is to ensure the participant complies with study procedures and to act as a collateral source who provides information about the participant’s current cognitive, functional, and behavioral status to the research team.^2–4^ Study partners must commit time and effort as study participants and provide insight into the cognitive status and function of the participant being observed longitudinally.^4^ They are usually spouses, adult children, or close friends of the participant.^5^

Attracting and retaining participants and study partners for AD research poses a consistent challenge for ADRCs.^6,7^ Recruitment and retention of AD trial participants and study partners have been cited as the biggest obstacles to developing new treatments, and attrition in clinical trials reduces data quality.^8,9^ One of the key barriers to participation is the burden associated with attending required annual study visits for both the participant and study partner. These visits can be time consuming and demanding for both participants and study partners and typically require traveling to the study site.^10–12^ While study partners do not take part in biomarker procedures such as positron emission tomography (PET) scans, magnetic resonance imaging (MRI), and lumbar punctures, which carry risks, they are required to attend annually and provide input on the participant. Perceived burden of participation is associated with lower attendance for study visits and lower retention in ADRC longitudinal cohorts.^10,13^ Moreover, the burdens associated with longitudinal participation can exacerbate inequities in recruitment, particularly of historically underrepresented groups.^6,14,15^ Existing ADRC longitudinal participants and study partners are primarily highly educated non-Hispanic Whites and are generally not representative of the broader US population.^16^

Because of these difficulties, there are ongoing efforts to enhance recruitment and participation in AD research, particularly among historically underrepresented groups, such as racial minorities and participants with diverse socio-economic status.^17,18^ To this end, the National Alzheimer’s Coordinating Center (NACC) recently advised all ADRCs to adopt a default policy of remunerating both research participants and their study partners for all participation-related activities in AD longitudinal research.^19^

The NACC guidance aims to help mitigate participation barriers, including financial obstacles, and enhance recruitment and retention, particularly among underrepresented groups. Ensuring that participants and their study partners are remunerated helps to remove financial barriers (e.g., time off work, transportation) to participation and provides some compensation for study burden, which may be particularly crucial for underrepresented groups.^19,20^ More generally, NACC sees remuneration as a “readily modifiable factor” that can promote fair and inclusive participation in research and ensure that no participant has out-of-pocket expenses to participate.^19^ In turn, remuneration can potentially improve the recruitment and retention of study subjects.^19^

### 1.1 | Remuneration practices in AD research

Most of the 36 ADRCs do not currently include remuneration to study partners in any way, and those that do generally do not evaluate the effect of remuneration on study partner perceptions of participation, recruitment, or retention.^21^ Thus, we have little empirical guidance about how current study partners at ADRCs will react to the adoption of remuneration as advised by NACC guidelines. Remuneration can serve multiple functions, including reimbursement for out-of-pocket expenses, compensation for time and study burdens, or incentivizing participation.^22,23^ On the positive side, remuneration could be seen as compensation for study burden and thus lower perceived costs of participating. It may also serve as an incentive to continue to participate, as it provides a new personal financial benefit from participation, representing a sign of respect for the contribution of study partners, and thereby raising study partner commitment to participation. The purpose of introducing remuneration for Knight ADRC study partners was to compensate them for time and effort.

On the negative side, remuneration may also raise ethical concerns such as the potential for undue influence when deciding whether to participate. There is a growing consensus, however, that remuneration for research participation is not only an acceptable ethical practice but perhaps even desirable or necessary given the current disparities present in AD recruitment and retention.^23–25^ Remuneration, by definition, is not coercive (coercion requires a threat). There is empirical and conceptual evidence that remuneration does not unduly influence participants or affect perceived risks.^23,25–27^ However, remuneration could conflict with the strong altruistic motivations for participation that are shared by the vast majority of participants and study partners in longitudinal cohort studies of AD.^10,13^ Introducing remuneration could negatively affect altruistic motivations by making participation seem transactional or by lowering trust in research.^26,28^ Altruism is not the sole motivation for research participation, and research participants have multiple reasons for participating that include personal benefits such as compensation, medical care, access to medical center resources, knowledge, or memory evaluations.^10,11,13,26,29^

More generally, the potential salutary effect of remuneration depends crucially on how study partners view it. For remuneration to reduce study partners’ perceived burden and serve as an incentive to continue, they would need to see remuneration as at least benign and ideally desirable and ethical, which has not been documented in AD research. Most current study partners in ADRC longitudinal cohort studies were not recruited with any expectation of remuneration but nonetheless chose to join. Given their strong altruistic motivations, they may not uniformly view remuneration as desirable, appropriate, or ethical for participation. Thus, we cannot assume that remuneration will have the desired effects on commitment, continued participation, and perceived burden among current study partners. If study participants do not view remuneration as ethical, then introducing remuneration could negatively affect their perceptions of study participation and ultimately their retention.

In this study, we surveyed current study partners at the Knight ADRC to learn about their views on remuneration for participation and how those views relate to their perceptions of study participation. At the time of data collection, the Knight ADRC did not remunerate study partners for their participation (their annual visit). The Knight ADRC provided remuneration to participants for some study procedures (e.g., lumbar puncture, MRI, PET scan), but not for the annual clinical and cognitive evaluation. Of note, the Knight ADRC implemented remuneration for study partners who attend the annual visit and remuneration for the participant’s annual clinical and cognitive evaluations after data for the present study were collected. The introduction of remuneration for study partners was not formally announced or publicized in advance of the survey. While remuneration can have multiple purposes,^19^ the goal of introducing remuneration for Knight ADRC participants was to compensate them for their time.

Collecting data on the study partner’s views of remuneration at this time point was beneficial for several reasons. First, we are aware of no other research on study partners’ views of remuneration or how these views might relate to their retention in the research study. These data therefore provide the first systematic evidence of how study partners in longitudinal cohort studies view remuneration. Given that the Knight ADRC has a sizeable number of self-identified Black (hereafter: Black) study partners (≈ 19% of the cohort), the survey also allowed us to examine the views of a historically underrepresented minority group. Second, these data provide information on study partners’ views of remuneration prior to remuneration being offered. Consequently, the survey data provide a basis for comparison in future research examining the effects of introducing remuneration on recruitment and retention. Given that NACC guidelines encourage ADRCs to remunerate study partners, the motivations of future study partners who are offered remuneration upon enrollment may differ from existing study partners, and these data serve as a baseline for future studies. Finally, this study contributes to the growing field of recruitment science in AD research, which aims to determine evidence-based methods for recruiting and retaining research participants.^30^

## 2 | METHODS

This study was part of a larger project focused on both study partners’ and study participants’ views of study remuneration as well as their motivations for, commitment to, and perceived burden of longitudinal participation.

### 2.1 | Participants

Survey respondents were recruited from the set of study partners enrolled in the Memory and Aging Project (MAP), the Knight ADRC longitudinal cohort at Washington University School of Medicine. To be eligible for this study, study partners had to be associated with a currently enrolled participant with a Clinical Dementia Rating (CDR) < 1, had consented to be contacted for other studies, and had participated in MAP in the past 10 years.^31^ Because the survey was also administered to participants, we excluded participants with CDR > 1 as they require assistance with informed consent, and their study partners.^32^ Only 4% of Knight ADRC participants are CDR > 1 and 96% of participants and their study partners were included in our survey sample. All current study partners as of March 2024 (*N* = 813) were invited to complete the survey. Table 1 presents demographic characteristics of survey respondents (*N* = 517) and the full set of current study partners. The sample is slightly older (2.7 years) than the full set of study partners but otherwise similar in demographics. This study was approved by the institutional review board of Washington University in St. Louis.

### 2.2 | Study procedures

Study partners completed a 15 minute survey instrument about their experience participating in MAP. Study partners were sent the invitation to complete the survey between March and May 2024. For study partners who had an e-mail address on file, we sent the survey invitation via e-mail, and they participated by completing the survey online in REDCap (Research Electronic Data Capture).^33,34^ For those who did not respond via e-mail or for whom we did not have an e-mail address on file, we sent the survey via postal mail. In total, *n* = 232 (45%) participated online, and *n* = 285 (55%) participated via postal mail. At the end of the survey, study partners could enter a raffle to win one of ten $100 gift cards. For analysis, we linked the completed surveys to demographic data from the Knight ADRC Biostatistics Core.

### 2.3 | Measures

Most of the survey instrument was derived from prior research using surveys of study partners in the same type of AD longitudinal study.^9,10,14^ First, we repeated a set of questions about motivations for participation that have been found to capture two fundamental dimensions: altruism and personal benefit. Personal benefit items addressed access to expertise, treatments, and knowledge about AD dementia as benefits of participation.^13^ Second, we included a validated shortened version of questions that comprise the Perceived Research Burden Assessment (PeRBA) index.^11,35,36^ Third, we included five original questions designed to capture the study partner’s commitment to continuing participation and three original questions designed to investigate their disposition toward remuneration for participation. The new items were reviewed by the Knight ADRC ethics committee, which consists of participants, study partners, and Knight ADRC clinical staff who provided input on the clarity and suitability of the items to help establish face validity of the items.^37^ The three payment items had a combined Flesch–Kincaid score of 11.2 (item 1: 5.2 grade level, item 2: 14.2 grade level, item 3: 13 grade level). Given the longstanding ethical concerns and debate about payment for participation and the potential implications for participation, one of the items asked about the ethicality of payment. We were interested in determining whether study partners considered payment unethical and, if so, whether that affected their commitment to participation.^23,25–27^ All items in the survey were rated on a Likert-type scale (1 = strongly disagree, 2 = disagree, 3 = neutral, 4 = agree, 5 = strongly agree). Finally, we included an item assessing study partners’ trust in medical researchers: “All things considered, how much do you trust medical researchers” (1 = not at all, 5 = a great deal). The full list of the survey items is presented in the supporting information.

The study partner survey contained the following instruction to clarify when items were asking about the study partner themselves or the participant: “This survey asks about your experiences being the study partner for an individual participating in AD research at Washington University’s MAP. We will refer to the individual taking part in MAP as your partner throughout this survey.” Each block of questions either explicitly referred to the study partner or included instructions regarding whether the respondent was being asked about their own views or that of their partner (e.g., “the next questions ask about your views”). Survey items regarding attitudes toward remuneration, commitment to the study, or perceived burden sought the views of study partners on their own remuneration or commitment, not that of the participant.

### 2.4 | Data analysis

All statistical analyses were conducted with STATA 19.0. We first conducted descriptive analyses on the three remuneration-related survey items by calculating means and standard deviations (SDs) and examining item distributions. Next, we conducted a principal component analysis (PCA) on these items to estimate the general disposition toward remuneration and generate component scores for study partners on that dimension. We determined the number of components to retain based on the Kaiser–Guttman criterion, the empirical Kaiser criterion, and parallel analysis.^38^ The parallel analysis was conducted with the paran procedure in STATA 19.0. We then used ordinary least squares regression to evaluate whether these component scores varied systematically by years of education, age, sex, or race in a multivariate analysis. We used Cronbach alpha to assess internal consistency reliability.

Second, we evaluated how the disposition to remuneration was associated with motivations for participation, perceived burden from participation, and commitment to participation. Mirroring the data analysis in past studies,^10,13^ we estimated PCA models for altruistic and personal benefit motivations and PeRBA study burden and generated component scores for study partners on each of these components. We also estimated a PCA model of the five new items associated with commitment to participation and generated component scores for study partners on the commitment component. Finally, we distinguished study partners who were negatively disposed toward remuneration for participation, which allows us to evaluate how the introduction of remuneration may adversely affect those study partners’ motivations, perceptions of burden, and commitment to study participation. Specifically, using difference-in-means tests with two-tailed tests, we compared the mean values on each of these components for study partners with negative dispositions toward remuneration to the mean value on these dimensions for study partners with neutral to positive dispositions toward remuneration. Note that the PCA analyses generate component scores that are normalized to a mean of zero and a SD of 1.

## 3 | RESULTS

**FIGURE 1:**
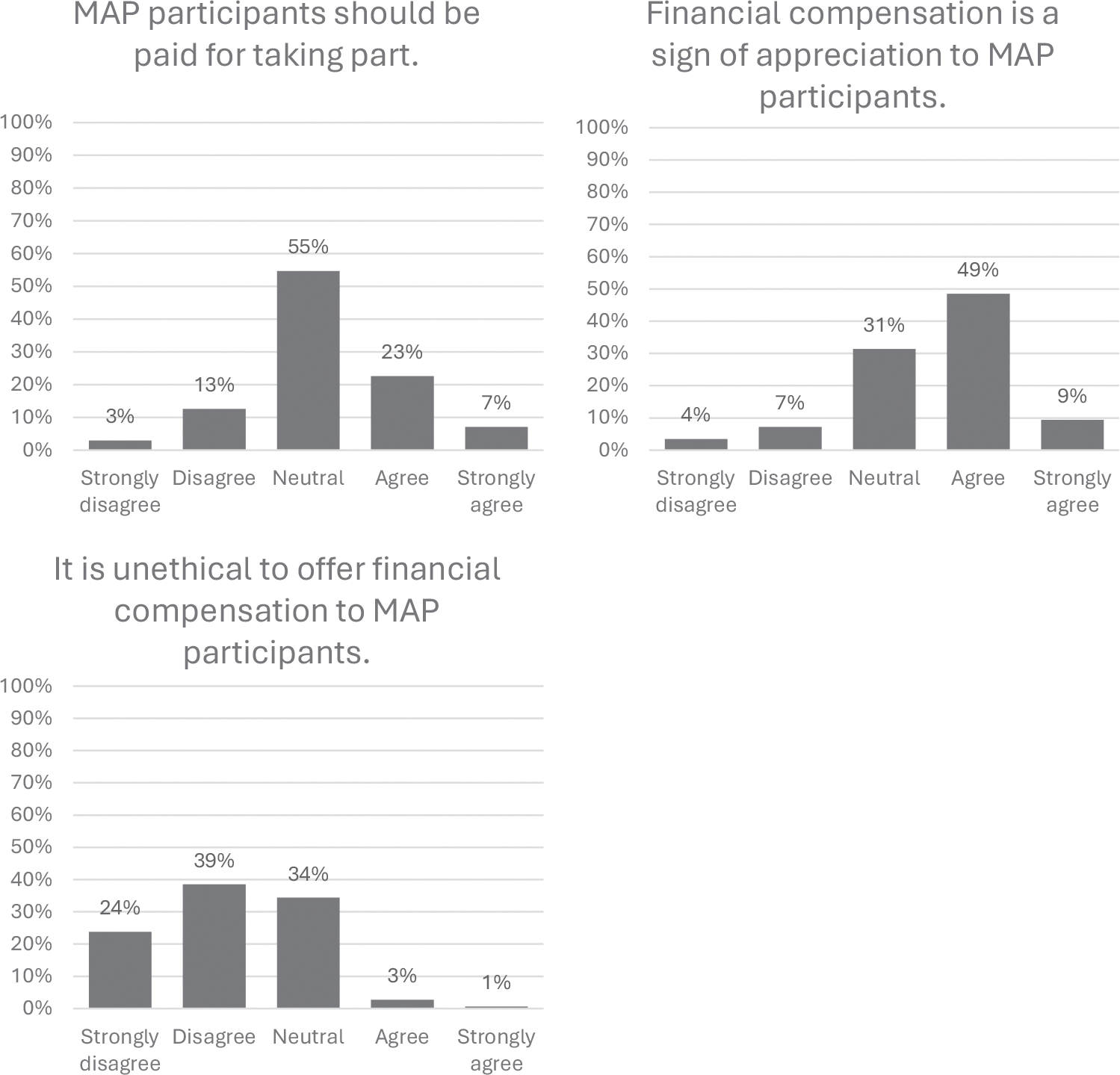
Distribution of responses to remuneration items. MAP, Memory and Aging Project.

Views of remuneration were assessed with three items: “MAP participants should be paid for taking part,” “Financial compensation is a sign of appreciation to MAP participants,” and “It is unethical to offer financial compensation to MAP participants.” See Figure 1 for the distribution of responses for each item. Examination of the means, medians, and distributions of the remuneration items showed that study partners felt largely neutral about whether MAP participants should be paid for taking part (Mean = 3.18, SD = 0.85, Median = 3). Study partners generally agreed that financial compensation is a sign of appreciation to MAP participants (Mean = 3.53, SD = 0.89, Median = 4). Furthermore, study partners generally disagreed with the idea that it is unethical to offer financial compensation to MAP participants (Mean = 2.18, SD = 0.84, Median = 2). Note that, for each question, some study partners expressed reservations about remuneration for participation (for instance, 16% disagreed that MAP participants should be paid for taking part). To the extent that the same study partners are registering these reservations across questions, the survey responses may reflect a general disposition toward remuneration.

To investigate that, Table 2 reports the PCA results of these three survey questions. The PCA strongly supports retention of a single component, based on the Kaiser–Guttman criterion, the empirical Kaiser criterion, and the parallel analysis (see Figure S1 in supporting information). Moreover, the loadings of the variables indicate the component captures variation in favorability toward remuneration. Specifically, responses agreeing that offering financial compensation is unethical are negatively associated with the component, while responses agreeing that financial compensation is a sign of appreciation were positively associated with the component. We then calculated a component score on the “favorability toward remuneration” index for each study partner. These scores distinguish study partners in terms of their favorability toward remuneration, with higher scores indicating a more positive disposition. Cronbach alpha for the items was *α* = 0.77, indicating good internal consistency reliability. Finally, as evidence of construct validity, we estimated the correlation between the study partners’ component scores on this index with their interest in entering a raffle to win a $100 gift card. All survey participants were asked whether they would like to be entered in the raffle, with 75% indicating they would and 25% indicating they would not. We would expect study partners’ favorability toward remuneration for study participation to be positively related to their interest in gaining a financial reward for participating in the survey about their participation. As hypothesized, the correlation was positive (point-biserial correlation coefficient and Pearson correlation coefficient = 0.29, *P* < 0.01).

**FIGURE 2:**
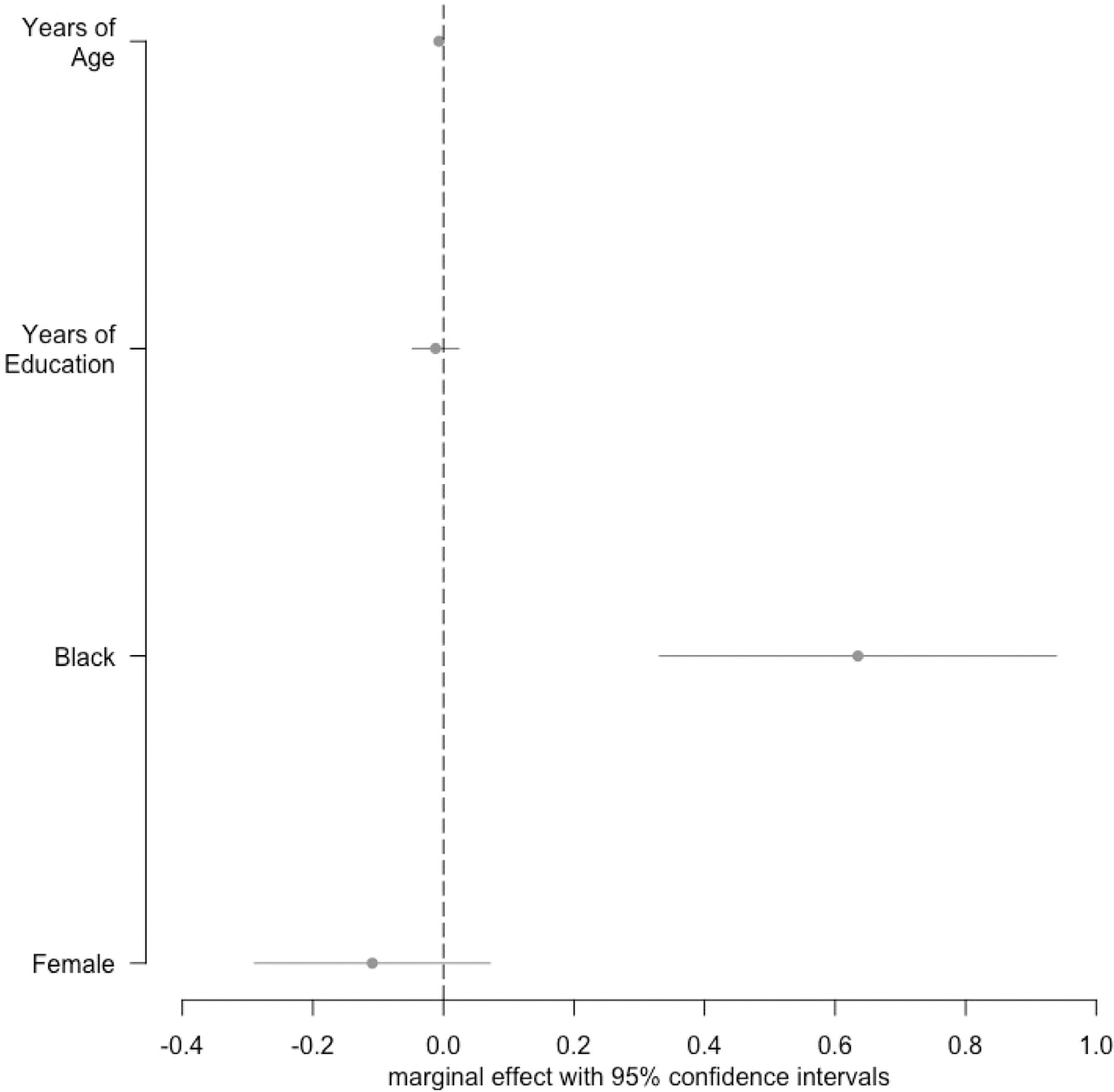
Effect of demographic characteristics on positive views toward remuneration. The marginal effects indicate the change in the favorability toward the remuneration index associated with differences in the demographic characteristics. For age and years of education, this reflects the difference in favorability associated with 1 year greater education or age. For self-reported race, this reflects a comparison of Black study partners relative to White study partners. For self-reported gender, this reflects a comparison of female relative to male. The figure is based on a multivariate ordinary least squares regression analysis including all four demographic characteristics.

Figure 2 reports the relationships of study partner age, years of education, race, and sex with favorability toward remuneration. The regression results indicated that Black study partners had significantly (*P* < 0.01) more positive views of remuneration than White study partners, even after controlling for age, years of education, and sex. The size of that effect was large (0.63) relative to the distribution of scores, which had a SD of 1.0.

We next evaluated how study partners’ favorability toward remuneration was related to several aspects of study participation. To capture motivations for study participation, we estimated a PCA of the same eight survey questions about reasons for participation used in previous studies of ADRC participants and of study partners.^9,13^ We replicated past findings, identifying two components: altruistic motivations and personal benefit motivations (see supporting information). As in past studies, we found that the vast majority of participants have strong altruistic motivations. For example, 97% of study partners either agreed or strongly agreed that they participate to benefit society. We then created component scores for study partners on both types of motivations. Similarly, we replicated a PCA of questions about study burden to generate a PeRBA component score for each study partner (analysis presented in supporting information). Finally, to measure commitment to participation, we conducted a PCA of study partners’ level of agreement with five new statements not administered in prior surveys: “I would like to continue participating in MAP as long as the health of my partner allows,” “I sometimes consider not continuing MAP,” “I can think of many things I would rather be doing than accompanying my partner to their scheduled visit,” “I would highly recommend participation as a study partner to my friends,” and “I am enthusiastic about the prospect of participating in future studies at the Knight ADRC.” Most study partners expressed positive views on these questions. For example, 94% of study partners agreed or strongly agreed that they would like to continue participating in MAP as long as their health allowed. Only 3% agreed or strongly agreed that they sometimes consider not continuing in MAP.

The PCA (Table 3) reveals that responses to these statements were structured by one component. The loadings of the items on the component were strong and in the expected directions (i.e., negative statements about commitment load in the opposite direction to positive statements). This indicated that the component was capturing a general commitment to study participation. Furthermore, as evidence of construct validity, the component scores were positively related (Pearson correlation coefficient = 0.42, *P* < 0.01) to study partners’ trust in medical researchers, indicated by their response to the question “All things considered, how much do you trust medical researchers?” (see supporting information). This provided additional validation that the component measures commitment to participation, as trust in medical researchers predicts study partner retention in AD studies.^39^

We wanted to evaluate whether study partners’ scores on these components differed between study partners with relatively negative dispositions about remuneration and study partners with more positive views on remuneration. This is important because although most individuals had positive views regarding remuneration, some respondents indicated reservations about remuneration. We used the remuneration component scores to distinguish these two groups. Study partners at or below the lowest decile on the remuneration component were classified as relatively negative, and the top 90% were considered relatively positive. This 10% cut point corresponds to the desired distinction in responses to the individual survey items that constitute the remuneration component. For example, for study partners above the lowest decile, almost none (<2%) disagreed or strongly disagreed with remuneration for participation, and 64% agreed or strongly agreed. In contrast, 79% of study partners in the lowest decile disagreed or strongly disagreed that MAP participants should be paid for participation, with the remaining 21% taking a neutral position.

**FIGURE 3:**
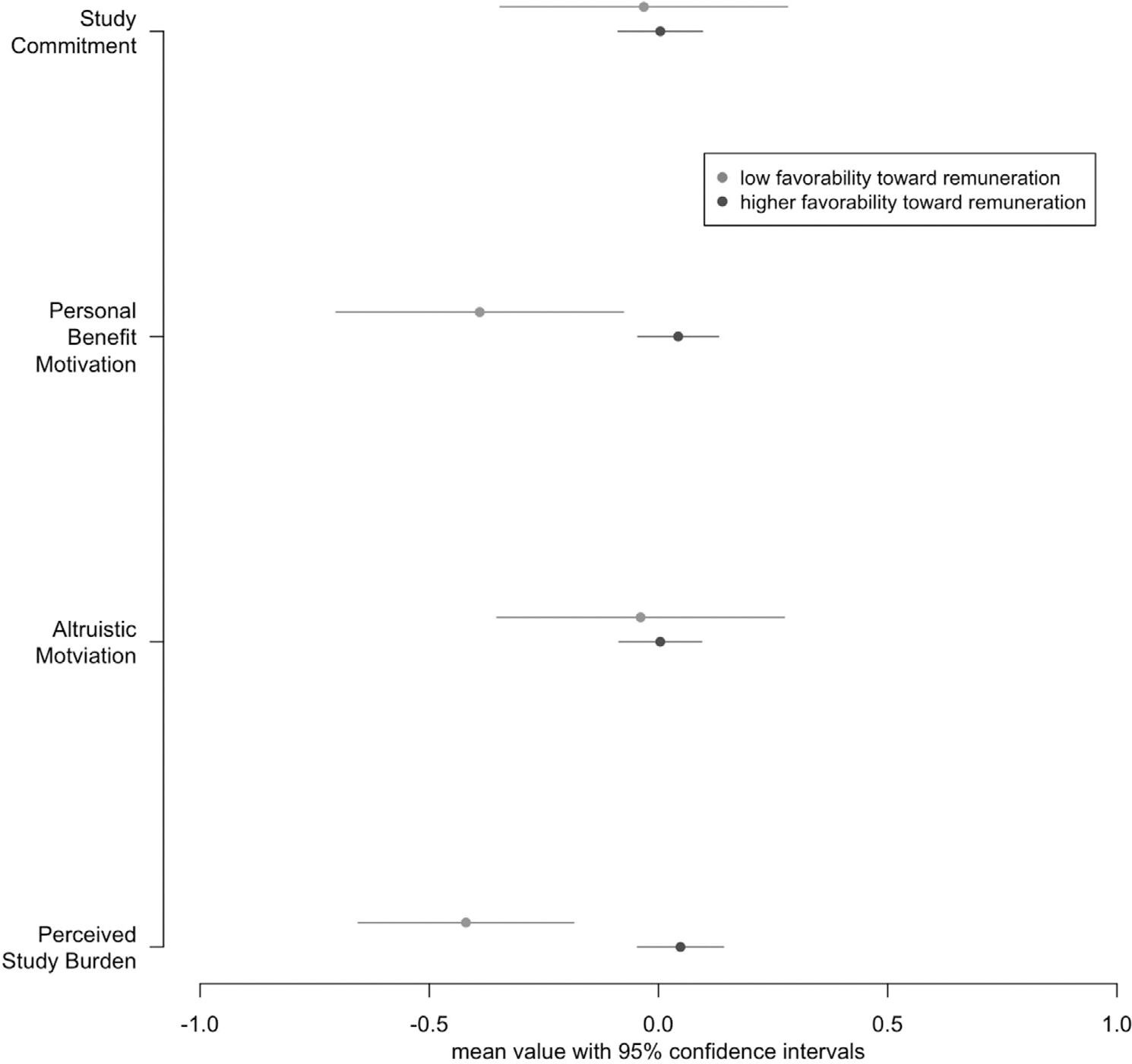
Differences in perceptions of participation related to positive views toward remuneration. The figure compares two groups of study partners: those in the lowest decile of favorability toward the remuneration index and those above the lowest decile on the favorability toward the remuneration index. For each type of perception about participation (e.g., altruistic motivation), the mean value and its 95% confidence interval on that index are reported for the two groups. The two groups are statistically different for perceived study burden (*P* < 0.01) and for personal benefit motivation (*P* < 0.01).

Figure 3 presents the mean score on each component of views on participation (e.g., altruistic motivation) for these two groups based on remuneration component scores. Study partners from the lowest decile on the remuneration component (i.e., negative disposition toward remuneration) had lower perceived study burden and lower personal benefit motivation than did study partners with more positive views on remuneration (*P* < 0.01). There was no difference (*P* > 0.79) between the two groups in terms of altruistic motivations or commitment to participation.

## 4 | DISCUSSION

The findings showed that study partners felt generally neutral to positive regarding remuneration for study participation. They generally agreed with the idea that remuneration is a sign of appreciation and felt largely neutral about the idea that they should be paid to take part. Study partners generally disagreed with the idea of compensation being unethical. The findings indicate that remuneration is perceived as an acceptable practice by most study partners but may not be expected or strongly favored. That said, some study partners expressed reservations about remuneration, with > 10% not considering remuneration appropriate or a sign of appreciation.

Furthermore, the analysis revealed that the survey items focused on remuneration were all measuring the same domain or underlying construct. This is important because it shows that study partners’ views toward remuneration are structured by a single dimension, even though the analysis of means and SDs shows variability in how each item was rated on average. This is an original finding, demonstrating that it is appropriate to analyze the items together as a single construct.

We found a substantively large and statistically significant difference between Black and White study partners in their favorability toward remuneration. Black study partners were substantially more favorable toward remuneration. Moreover, in the group of study partners with the most negative disposition toward payment, there were no Black study partners. This suggests that introducing remuneration for study visits will be viewed particularly favorably by Black study partners. Given that one motivation for the NACC recommendation to provide such remuneration is to improve recruitment and retention of historically underrepresented groups, this is an important finding.

Our findings provide important evidence as to whether introducing remuneration to current study partners will have the desired salutary effect on perceptions of participation and retention and avoid potential problems. For remuneration to improve retention, study partners need to view remuneration favorably or, at least, neutrally. We find that, in general, they do. However, some study partners had negative dispositions and could respond adversely to the introduction of compensation. All participants should be offered the option to decline compensation should they wish, which is an option at the Knight ADRC. If those with negative views were also relatively low on commitment, introducing remuneration could undermine their retention. Our analysis indicates this is likely not a problem for current study partners. We developed and validated an original measure of commitment to participation based on multiple survey questions. We found that study partners with the most negative views on remuneration shared the same high levels of commitment as the study partners with more positive views on remuneration.

Another potential salutary effect of introducing remuneration is that it would satisfy study partners’ goals in terms of deriving personal benefits from participation. That is a common motivation for participation among the surveyed study partners.^13^ However, if the subset of study partners with negative views on remuneration are also highly motivated by personal benefits, this salutary effect may be limited. Our findings indicate this is not a concern for introducing remuneration to study partners. Study partners with negative views on remuneration expressed relatively low personal benefit motivations compared to study partners with more positive views on remuneration.

A final potential benefit to introducing remuneration is that it could increase study partners’ willingness to accept study-related burdens and thus reduce their perceptions of study burden.^19^ This depends on whether those with a high study burden view remuneration favorably and whether those with relatively negative views on remuneration perceive a relatively low burden. Our results suggest this is the case. The study partners with the most negative views on remuneration had distinctly lower levels of perceived study burden than those with relatively positive views on remuneration. Put differently, for those perceiving high burden, remuneration is viewed favorably, while those who view remuneration less favorably do not perceive high study burden and plausibly may not view remuneration as necessary or favorable.

A final potential concern with introducing remuneration is that it could adversely affect study partners’ altruistic goals of participation. The majority of study partners surveyed expressed very strong altruistic motivations for study participation.^13^ If study partners with negative views on remuneration also had stronger altruistic motivations compared to those who have positive or neutral views regarding remuneration, this would suggest that remuneration may negatively affect altruistic goals. Here again, our findings are reassuring. Our results indicate that study partners who held more negative views on remuneration did not express higher altruistic motivations compared to those with positive or neutral views on remuneration. Taken together, these findings suggest that following the NACC guidance to remunerate study partners is unlikely to cause harm and may help with study partner retention.

The current study had a number of strengths. To our knowledge, this is the first study focused on study partners’ views of remuneration. The requirement to have a study partner to enroll in AD longitudinal cohorts is a barrier to participation,^12,40^ so determining study partners’ views regarding remuneration is important for understanding strategies that could enhance study partner retention, satisfaction, or goal attainment from participation. Our findings suggest that introducing remuneration is unlikely to harm retention or be perceived negatively by study partners already enrolled in AD longitudinal research.^26,41^ Additionally, the response rate for the survey was very high (64%) and yielded a large and diverse sample of study partners.

A further strength of our study is that it provides empirical data on the attitudes of study partners who are enrolled in research regarding remuneration. There is an existing literature on recruitment and retention to AD research which often addresses ways to increase representation of historically underrepresented individuals.^16,17,41–43^ Remuneration has been cited as one way to increase recruitment and retention of AD research participants, particularly for those who are underrepresented.^22^ However, there is a lack of rigorous empirical evidence regarding the impact of any strategy, including remuneration, on actual recruitment or retention, leaving major scientific gaps.^41^ Our team conducted one experimental vignette study on stated willingness to enroll in an AD longitudinal cohort, oversampled for self-identified Black and Hispanic participants, which found that payments of $50 to $100 increased stated willingness to enroll among all participants, did not alter perceptions of risk, and lowered perceived burden.^26^ However, this study was about stated willingness, not actual enrollment. Future studies should examine how remuneration affects actual enrollment, which remains unknown and which this study does not address.

There were also limitations to the current study. While the sample was large, the data were collected from one ADRC in the Midwestern United States. The survey was specific to study partners taking part in longitudinal observational research, which requires ongoing commitment but carries less than minimal risk. These findings may not generalize to study participants whose attitudes may differ and vary depending on the risk level of the study. Survey respondents did not differ from the Knight ADRC study partner population as a whole in terms of education, sex, race, or relationship to the participant, but they were significantly older (Table 1). The difference in age is unlikely to affect our findings given age was not correlated with any of the reported study outcomes (Figure 2). Our sample had a mean education of 16.2 years. Knight ADRC study partners are similarly representative to those reported by Horn et al.^13,44^ However, it’s possible that Knight ADRC study partners differ from those at other ADRCs or in AD studies outside of the United States.^16,44^ Also, while 16% of the overall study partner population at the Knight ADRC was Black, only 9% of the survey sample was Black. More diverse samples, such as those with varying education, race, or ethnicity, may perceive remuneration differently, or there may be a group of people for whom remuneration is an important motivator who were not well represented in the current study. For example, more highly educated individuals tend to have higher incomes and face fewer obstacles associated with study visits, and thus may be less motivated by remuneration than someone in a lower socioeconomic status who may have greater difficulty taking time off work or traveling to the study visit.^6,14,15^ Future research should examine views of remuneration in more diverse samples and across a broader range of remuneration-related concerns than the three questions provided here. Survey items will need to be modified to ensure they are suitable for those with lower health literacy, given the grade reading level of current items was high but was unlikely to affect face validity given the high education of this survey sample.^37^ Future research should also examine the psychometric properties of these survey items in other, more diverse, samples and evaluate the reproducibility of our survey items. This may reveal a more complex structure to views on remuneration and participation than the single dimension reported here. Similarly, because our survey only included a general question about whether remuneration for participation was ethical, our analysis did not investigate or compare the potentially many ethical concerns that remuneration might raise for study partners. In addition, future research involving qualitative interviews with those who had positive and negative views regarding remuneration would add depth to quantitative findings. Last, data were collected online using REDCap and also via paper surveys mailed to study partners. One might be concerned that the mode of survey administration might introduce unwanted variability or method effects, but we found no significant differences between those who completed the survey in Redcap versus mail surveys on perceived burden, altruism, personal benefits, commitment, or views on remuneration (see supporting information).

## 5 | CONCLUSIONS

Study partners’ views of remuneration were found to be neutral to favorable and suggest that remunerating study partners is unlikely to cause harm or affect altruistic motivations for participating in AD research. Remuneration seems to be perceived as an acceptable practice by study partners but may not be expected or strongly favored. These findings contribute to the growing field of recruitment science, demonstrating study partners’ views of remuneration and how those views are related to other important barriers (e.g., perceived burden), motivators (e.g., altruism and perceived personal benefits), and their commitment to participating in AD research. Determining evidence-based methods for recruitment in AD research is imperative for improving or introducing strategies that ultimately help advance clinical research and develop new treatments. Understanding what motivates individuals to participate in AD research and how to overcome barriers to participation using data-driven tactics will help achieve recruitment and retention goals of ADRCs.^6,7^

## Supplementary Material

Support 1

Support 2 Figures

ICJME

SUPPORTING INFORMATION

Additional supporting information can be found online in the Supporting Information section at the end of this article.

## Figures and Tables

**TABLE 1:** Study partner characteristics.

Characteristic	Knight ADRC study partner population (*N* = 813)	Survey sample (*n* = 517)
Age (mean)	66.1 years	68.8 years
Education (mean)	16.0 years	16.2 years
**Sex**		
Female	516 (64%)	316 (61%)
Male	296 (36%)	200 (39%)
Other	1 (<1%)	1 (<1%)
**Race**		
Black	133 (16%)	49 (9%)
White	670 (82%)	461 (89%)
Other	10 (1%)	7 (1%)
**Relationship to participant**		
Spouse	411 (51%)	308 (60%)
Child	196 (24%)	83 (16%)
Friend	102 (13%)	63 (12%)
Sibling	62 (8%)	35 (7%)
Other family (e.g., cousin, aunt, grandchild)	38 (5%)	24 (5%)
Other	4 (<1%)	4 (1%)

*Note:* Sixteen years of education is equivalent to a bachelor’s degree in the United States. One-sample *t* tests indicated that the sample’s age is significantly older than the population’s age, *t*(515) = 4.89, *P* < 0.001, but there was no significant difference in education. Chi-squared goodness-of-fit tests indicated no significant differences between the population and the sample on sex, race, or relationship to the participant.

Abbreviation: ADRC, Alzheimer’s Disease Research Center.

**TABLE 2:** Principal component analysis (PCA) of remuneration items.

Item	Component loading
Financial compensation is a sign of appreciation to MAP participants.	0.86
MAP participants should be paid for taking part.	0.81
It is unethical to offer financial compensation to MAP participants.	−0.81
Eigenvalue of component	2.05
Reference eigenvalue (empirical Kaiser criterion)	1.16
% variance explained	68.21
Cronbach alpha	0.77

*Note:* The table presents the loadings for the first component. The PCA extracted three components, but the eigenvalues (0.54 and 0.41) for the other two components were well below the Kaiser-Guttman criterion of 1.0. The empirical Kaiser criterion recommends retaining only components with an eigenvalue > 1.0 and greater than its reference eigenvalue.^38^

Abbreviations: ADRC, Alzheimer’s Disease Research Center; MAP, Memory and Aging Project.

**TABLE 3:** Principal component analysis of commitment items.

Item	Component loading
I would like to continue participating in MAP as long as the health of my partner allows.	0.74
I sometimes consider not continuing in MAP.	−0.74
I can think of many things I would rather be doing than accompanying my partner to their scheduled visits.	−0.67
I would highly recommend participation as a study partner to my friends.	0.80
I am enthusiastic about the prospect of participating in future studies at the Knight ADRC.	0.80
Eigenvalue of component	2.82
% variance explained	56.45
Cronbach alpha	0.80

*Note:* Only one component was extracted that had an eigenvalue > 1.00; the next highest eigenvalue was 0.82.

Abbreviations: ADRC, Alzheimer's Disease Research Center; MAP, Memory and Aging Project.

## Data Availability

Knight ADRC data can be requested through the online request portal on the Knight ADRC website: https://knightadrc.wustl.edu/professionals-clinicians/request-center-resources/

## RESEARCH IN CONTEXT

### 1. Systematic review:

The authors reviewed literature on recruitment, retention, and views of study partners on remuneration in Alzheimer’s disease (AD) research.

### 2. Interpretation:

New guidelines recommend remuneration for all AD research participants and study partners. Findings suggest that study partners feel neutral to positive about remuneration. There were no differences in views on remuneration based on sex or education; however, self-identified Black study partners had more favorable views on remuneration. Study partners with the least favorable views on remuneration perceived a lower participation burden and were less motivated by the perceived personal benefits of participating, but all study partners were motivated by altruism and committed to continued participation. Remuneration appears to be perceived as an acceptable practice by study partners but may not be expected or strongly favored.

### 3. Future directions:

Future research should evaluate the impact of remuneration on the recruitment and retention of more diverse AD research participants and study partners.

## HIGHLIGHTS

- We examined study partners’ views on remuneration for Alzheimer’s disease research participation.
- Study partners felt largely neutral to positive about remuneration.
- All study partners were highly motivated by altruism and committed to continued participation.
- Black study partners viewed remuneration more positively than White study partners.
- Remuneration may improve recruitment and retention of historically underrepresented groups.

## References

[1] National Institute on Aging: Funded Alzheimer’s Disease Reseach Centers. 2024. https://www.nia.nih.gov/research/adc

[2] Nosheny RL, Jin C, Neuhaus J, Study partner-reported decline identifies cognitive decline and dementia risk. Ann Clin Transl Neurol. 2019;6(12):2448–2459. 31721455 10.1002/acn3.50938 PMC6917311

[3] Ryan MM, Grill JD, Gillen DL, Alzheimer’s Disease Neuroimaging I. Participant and study partner prediction and identification of cognitive impairment in preclinical Alzheimer’s disease: study partner vs. participant accuracy. Alzheimers Res Ther. 2019;11(1):85. 31627738 10.1186/s13195-019-0539-3 PMC6800492

[4] Largent EA, Karlawish J, Grill JD. Study partners: essential collaborators in discovering treatments for Alzheimer’s disease. Alzheimers Res Ther. 2018;10(1):101. 30261910 10.1186/s13195-018-0425-4 PMC6161465

[5] Cary MS, Rubright JD, Grill JD, Karlawish J. Why are spousal caregivers more prevalent than nonspousal caregivers as study partners in AD dementia clinical trials?. Alzheimer Dis Assoc Disord. 2015;29(1):70–74. 24805971 10.1097/WAD.0000000000000047 PMC4223007

[6] Grill JD, Galvin JE. Facilitating Alzheimer disease research recruitment. Alzheimer Dis Assoc Disord. 2014;28(1):1–8. 24322484 10.1097/WAD.0000000000000016 PMC3945167

[7] Grill JD, Karlawish J. Addressing the challenges to successful recruitment and retention in Alzheimer’s disease clinical trials. Alzheimers Res Ther. 2010;2(6):34. 21172069 10.1186/alzrt58 PMC3031880

[8] Pharmaceutical Research and Manufacturers of America. Medicines in Development: Alzheimer’s Disease. PhRMA; 2013.

[9] Little Roderick J, D’Agostino R, Cohen Michael L, The prevention and treatment of missing data in clinical trials. N Engl J Med. 2012;367(14):1355–1360. 23034025 10.1056/NEJMsr1203730 PMC3771340

[10] Gabel M, Bollinger RM, Coble DW, Retaining participants in longitudinal studies of Alzheimer’s disease. J Alzheimers Dis. 2022;87(2):945–955. 35404282 10.3233/JAD-215710 PMC9673904

[11] Gabel M, Bollinger RM, Knox M, Perceptions of Research burden and retention among participants in ADRC cohorts. Alzheimer Dis Assoc Disord. 2022;36(4):281–287. 35796752 10.1097/WAD.0000000000000514 PMC9712497

[12] Black BS, Taylor HA, Rabins PV, Karlawish J. Study partners perform essential tasks in dementia research and can experience burdens and benefits in this role. Dementia. 2016;17(4):494–514. 10.1177/1471301216648796 PMC5107353 27179001

[13] Bollinger RM, Gabel M, Coble DW, Retention of study partners in longitudinal studies of Alzheimer disease. J Alzheimers Dis. 2023;94(1):189–199. 37212114 10.3233/JAD-230079 PMC10515740

[14] Zhou Y, Elashoff D, Kremen S, Teng E, Karlawish J, Grill JD. African Americans are less likely to enroll in preclinical Alzheimer’s disease clinical trials. Alzheimers Dement. 2017;3(1):57–64. 10.1016/j.trci.2016.09.004 PMC5651355 29067319

[15] Grill JD, Monsell S, Karlawish J. Are patients whose study partners are spouses more likely to be eligible for Alzheimer’s disease clinical trials?. Dement Geriatr Cogn Disord. 2012;33(5):334–340. 22759982 10.1159/000339361 PMC3477789

[16] Arce Renteria M, Mobley TM, Evangelista ND, Representativeness of samples enrolled in Alzheimer’s disease research centers. Alzheimers Dement. 2023;15(2):e12450. 10.1002/dad2.12450 PMC10242202 37287650

[17] Alzheimer’s Association. Special Report: Race, Ethnicity and Alzheimer’s in America. Alzheimer’s Association; 2021;17:71–104.

[18] Babulal GM, Quiroz YT, Albensi BC, Perspectives on ethnic and racial disparities in Alzheimer’s disease and related dementias: update and areas of immediate need. Alzheimers Dement. 2019;15(2):292–312. 30555031 10.1016/j.jalz.2018.09.009 PMC6368893

[19] National Alzheimer’s Coordinating Center. Guidance on Offers of Payment to Research Participants. 2023. https://naccdata.org/adrc-resources/best-practices

[20] Persad G, Fernandez Lynch H, Largent E. Differential payment to research participants in the same study: an ethical analysis. J Med Ethics. 2019;45(5):318–322. doi: 10.1136/medethics-2018-105140 30846490

[21] Gabel M, Bekena S, O’Meara M, Current practices by Alzheimer’s Disease Research Centers to remunerate research participants. Alzheimers Dement. 2025;21(2):e14542. 39868808 10.1002/alz.14542 PMC11851152

[22] National Alzheimer’s Coordinating Center. Guidance on Offers of Payment to Research Participants. ADRC Best Practices; 2023.

[23] Gelinas L, Largent EA, Cohen IG, Kornetsky S, Bierer BE. A Framework for Ethical Payment to Research Participants. N Engl J Med. 2018;378(8):766–771. doi: 10.1056/NEJMsb1710591 29466147

[24] Grady C Payment of clinical research subjects. J Clin Invest. 2005;115(7):1681–1687. 16007244 10.1172/JCI25694 PMC1159153

[25] Largent EA, Fernandez Lynch H. Paying research participants: regulatory uncertainty, conceptual confusion, and a path forward. Yale J Health Policy Law Ethics. 2017;17(1):61–141. 29249912 PMC5728432

[26] Gabel M, Denny A, Llibre-Guerra J, Morris JC, Phillips J, Vaidyanathan A. Remuneration and recruitment of study participants for AD cohort studies from the general public and from minority communities. Alzheimer Dis Assoc Disord. 2023;37(2):107–112. 37145978 10.1097/WAD.0000000000000556 PMC10239367

[27] Largent EA, Lynch HF. Paying research participants: the outsized influence of “undue influence”. IRB. 2017;39(4):1–9. PMC5640154 29038611

[28] Schneider FH, Campos-Mercade P, Meier S, Pope D, Wengström E, Meier AN. Financial incentives for vaccination do not have negative unintended consequences. Nature. 2023;613(7944):526–533. 36631607 10.1038/s41586-022-05512-4 PMC9833033

[29] Largent EA, Eriksen W, Barg FK, Greysen SR, Halpern SD. Participants’ perspectives on payment for research participation: a qualitative study. Ethics Hum Res. 2022;44(6):14–22. 10.1002/eahr.500147 PMC9631331 36316972

[30] National Institute on Aging. Together We Make the Difference: National Strategy for Recruitment and Participation in Alzheimer’s and Related Dementias Clinical Research. 2018. 10.18865/ed.30.S2.705 PMC7683031 33250617

[31] Morris JC. The Clinical Dementia Rating (CDR): current version and scoring rules. Neurology. 1993;43(11):2412–2414. doi: 10.1212/wnl.43.11.2412-a 8232972

[32] Buckles VD, Powlishta KK, Palmer JL, Understanding of informed consent by demented individuals. Neurology. 2003;61(12):1662–1666. 14694026 10.1212/01.wnl.0000098933.34804.fc

[33] Harris PA, Taylor R, Thielke R, Payne J, Gonzalez N, Conde JG. Research electronic data capture (REDCap)—A metadata-driven methodology and workflow process for providing translational research informatics support. J Biomed Inform. 2009;42(2):377–381. 18929686 10.1016/j.jbi.2008.08.010 PMC2700030

[34] Harris PA, Taylor R, Minor BL, The REDCap consortium: building an international community of software platform partners. J Biomed Inform. 2019;95:103208. 31078660 10.1016/j.jbi.2019.103208 PMC7254481

[35] Lingler JH, Schmidt KL, Gentry AL, Hu L, Terhorst LA. A new measure of research participant burden: brief report. J Empir Res Hum Res Ethics. 2014;9(4):46–49. 10.1177/1556264614545037 PMC4487419 26125079

[36] Keleman AA, Chang CH, Bollinger RM, Lingler JH, Gabel M, Stark SL. Psychometric evaluation of the Perceived Research Burden Assessment (PeRBA) in longitudinal studies of Alzheimer disease using Rasch analysis. Alzheimer Dis Assoc Disord. 2023;37(1):28–34. 36251929 10.1097/WAD.0000000000000532 PMC9974570

[37] DeVon HA, Block ME, Moyle-Wright P, A psychometric toolbox for testing validity and reliability. J Nurs Scholarsh. 2007;39(2):155–164. 17535316 10.1111/j.1547-5069.2007.00161.x

[38] Auerswald M, Moshagen M. How to determine the number of factors to retain in exploratory factor analysis: a comparison of extraction methods under realistic conditions. Psychol Methods. 2019;24(4):468–491. 30667242 10.1037/met0000200

[39] Stites SD, Turner RS, Gill J, Research attitudes questionnaire scores predict Alzheimer’s disease clinical trial dropout. Clin Trials. 2021;18(2):237–244. 33426901 10.1177/1740774520982315 PMC8009810

[40] Black BS, Taylor H, Rabins PV, Karlawish J. Researchers’ perspectives on the role of study partners in dementia research. Int Psychogeriatr. 2014;26(10):1649–1657. 24990196 10.1017/S1041610214001203 PMC4349344

[41] Gilmore-Bykovskyi AL, Jin Y, Gleason C, Recruitment and retention of underrepresented populations in Alzheimer’s disease research: a systematic review. Alzheimers Dement. 2019;5:751–770. 10.1016/j.trci.2019.09.018 PMC6944728 31921966

[42] Mozersky J, Dimtsu Assfaw A, Balls Berry JE, Enhancing participation of historically minoritized groups in Alzheimer disease and related dementias research: National Conference Report. Alzheimers Dement. 2025;21(5):e70168. 40342234 10.1002/alz.70168 PMC12060131

[43] Savold J, Cole M, Thorpe RJ Jr. Barriers and solutions to Alzheimer’s disease clinical trial participation for Black Americans. Alzheimers Dement. 2023;9(3):e12402. 10.1002/trc2.12402 PMC10318422 37408664

[44] Horn O, Hickey RE, Nietert PJ, Benitez A, Aghamoosa S. Study partner characteristics moderate the prediction of cognitive decline in aging. Alzheimers Dement. 2025;21(5):e70240. 40356018 10.1002/alz.70240 PMC12069006

