# Supplementary material for: Study partners’ views on remuneration in longitudinal Alzheimer’s disease research
Source: Alzheimers Dement Behav Socioecon Aging. Author manuscript; Available in PMC 2025 Dec 6. (PMC12680024; doi:10.1002/bsa3.70048)
Supplement: Support 1 [file NIHMS2123218-supplement-Support_1.docx]

**Study Partners’ Attitudes toward Remuneration in Alzheimer Disease Research**

**Supplemental materials**

This supplemental file has four parts:

1. Survey items
2. Means and standard deviations of all items
3. Principal Component Analyses replicating past results: perceived burden, altruism, and personal benefits
4. Comparison of participants who completed the survey online vs. mail

**Part 1: Survey items**

This survey asks about your experiences being the study partner for an individual participating in Alzheimer disease research at Washington University’s Memory and Aging Project (MAP). **We will refer to the individual taking part in MAP as your partner throughout this survey.** MAP is the clinical research office of the Knight Alzheimer Disease Research Center (ADRC) where you attend your annual study visit. There are no right or wrong answers to the following survey questions; we simply wish to know your thoughts and opinions. Your responses are anonymous and confidential. The researchers who conduct your annual visits will not know who participated in this survey.

**For each statement, please indicate how much you agree or disagree.**

1 = Strongly disagree

2 = Disagree

3 = Neutral

4 = Agree

5 = Strongly agree

**Perceived burden items**

1. I feel that study visits are too frequent.
2. I feel that study visits are too long.
3. I feel that MAP visits take away from time with my friends and family.
4. I feel that researchers call or contact me or my partner too often.
5. I worry that my personal information might not be kept private.
6. I feel that my partner became emotionally upset by the study procedures.
7. I worry that my partner may be physically harmed by some of the research procedures.
8. I have had second thoughts about my decision to participate in MAP.
9. I feel that the study takes too much time away from my or my partners job.
10. I feel that it costs too much to transport my partner to the research center.
11. My partner experienced side effects from the research procedures.
12. I feel that my partners health got worse while participating in MAP.

**Altruism items**

1. I support my partners participation in MAP to advance research about Alzheimer disease.
2. I support my partners participation in MAP to benefit society.
3. I support my partners participation in MAP to benefit future generations of my family.

**Personal benefits items**

1. I support my partners participation in MAP because I have concerns about their memory.
2. I support my partners participation in MAP to gain access to support at the medical center (advice and expertise).
3. I support my partners participation in MAP to learn more about Alzheimer disease and other dementias.
4. I support my partners participation in MAP to have access to future state-of-the-art treatments and information.
5. I support my partners participation in MAP because they enjoy spending time with the staff.

**Commitment items**

1. I would like to continue participating in MAP as long as the health of my partner allows.
2. I sometimes consider not continuing in MAP.
3. I can think of many things I would rather be doing than accompanying my partner to their scheduled visits.
4. I would highly recommend participation (as a study participant/partner) to my friends.
5. I am enthusiastic about the prospect of participating in future studies at the Knight ADRC.

**Remuneration items**

1. MAP participants should be paid for taking part.
2. Financial compensation is a sign of appreciation to MAP participants.
3. It is unethical to offer financial compensation to MAP participants.

**Trust in medical researchers item**

The next questions ask about your views on medical researchers. For each statement, please choose the response that best represents your opinion.

1 = Not at all

2 = A little

3 = Some

4 = Quite a bit

5 = A great deal

1. All things, considered, how much do you trust medical researchers?

**Part 2:** **Means and standard deviations of all survey items**

**Table S1. Means and standard deviations of all survey items**

| **Survey items** | **Mean** | **SD** |
| --- | --- | --- |
| Perceived burden items |  |  |
| 1. I feel that study visits are too frequent. | 2.19 | .774 |
| 1. I feel that study visits are too long. | 2.23 | .809 |
| 1. I feel that MAP visits take away from time with my friends and family. | 1.75 | .727 |
| 1. I feel that researchers call or contact me or my partner too often. | 1.81 | .715 |
| 1. I worry that my personal information might not be kept private. | 1.89 | .890 |
| 1. I feel that my partner became emotionally upset by the study procedures. | 1.58 | .715 |
| 1. I worry that my partner may be physically harmed by some of the research procedures. | 1.66 | .856 |
| 1. I have had second thoughts about my decision to participate in MAP. | 1.52 | .705 |
| 1. I feel that the study takes too much time away from my or my partners job. | 1.48 | .654 |
| 1. I feel that it costs too much to transport my partner to the research center. | 1.56 | .709 |
| 1. My partner experienced side effects from the research procedures. | 1.60 | .817 |
| 1. I feel that my partners health got worse while participating in MAP. | 1.49 | .704 |
| Altruism items |  |  |
| 1. I support my partners participation in MAP to advance research about Alzheimer disease. | 4.81 | .513 |
| 1. I support my partners participation in MAP to benefit society. | 4.77 | .560 |
| 1. I support my partners participation in MAP to benefit future generations of my family. | 4.66 | .679 |
| Personal benefits items |  |  |
| 1. I support my partners participation in MAP because I have concerns about their memory. | 3.14 | 1.357 |
| 1. I support my partners participation in MAP to gain access to support at the medical center (advice and expertise). | 3.72 | 1.205 |
| 1. I support my partners participation in MAP to learn more about Alzheimer disease and other dementias. | 4.30 | .857 |
| 1. I support my partners participation in MAP to have access to future state-of-the-art treatments and information. | 4.20 | .990 |
| 1. I support my partners participation in MAP because they enjoy spending time with the staff. | 3.28 | 1.050 |
| Commitment items |  |  |
| 1. I would like to continue participating in MAP as long as the health of my partner allows. | 4.37 | .671 |
| 1. I sometimes consider not continuing in MAP. | 1.88 | .771 |
| 1. I can think of many things I would rather be doing than accompanying my partner to their scheduled visits. | 2.23 | .940 |
| 1. I would highly recommend participation (as a study participant/partner) to my friends. | 4.05 | .809 |
| 1. I am enthusiastic about the prospect of participating in future studies at the Knight ADRC. | 3.81 | .872 |
| Remuneration items |  |  |
| 1. MAP participants should be paid for taking part. | 3.18 | .850 |
| 1. Financial compensation is a sign of appreciation to MAP participants. | 3.53 | .890 |
| 1. It is unethical to offer financial compensation to MAP participants. | 2.18 | .845 |
| Trust in medical researchers item |  |  |
| 1. All things, considered, how much do you trust medical researchers? | 4.31 | .668 |

*Note*. Items 1-28 were rated on a Likert-type scale (1=*strongly disagree*, 2=*disagree,* 3=*neutral,* 4=*agree,* 5=*strongly agree*). Item 29 was rated on a 5-point scale (1=*not at all*, 2=*a little,* 3=*some,* 4=*quite a bit,* 5=*a great deal*).

**Part 3: Principal Component Analyses replicating past results: perceived burden, altruism, and personal benefits**

We conducted a series of Principal Components Analyses (PCA) in order to determine whether the perceived burden, altruism, and personal benefits items replicate the structure seen in past literature and could be analyzed together each as their own measure. The PCA analyses are presented here, while the PCAs for the commitment and remuneration items are presented in the main article.

**Table S2. Principal Component Analysis (PCA) of Perceived Burden Items.**

| **Item** | **Component loading** |
| --- | --- |
| I feel that study visits are too frequent. | 0.62 |
| I feel that study visits are too long. | 0.63 |
| I feel that MAP visits take away from time with my friends and family. | 0.74 |
| I feel that researchers call or contact me or my partner too often. | 0.76 |
| I worry that my personal information might not be kept private. | 0.64 |
| I feel that my partner became emotionally upset by the study procedures. | 0.70 |
| I worry that my partner may be physically harmed by some of the research procedures. | 0.68 |
| I have had second thoughts about my decision to participate in MAP. | 0.73 |
| I feel that the study takes too much time away from my or my partners job. | 0.81 |
| I feel that it costs too much to transport my partner to the research center. | 0.74 |
| My partner experienced side effects from the research procedures. | 0.66 |
| I feel that my partners health got worse while participating in MAP. | 0.71 |
| Eigenvalue of component | 5.96 |
| % variance explained | 49.63% |
| Cronbach’s alpha | 0.90 |

*Note*. Two components were extracted that had an eigenvalue above the standard threshold of 1.00. However, visual examination of the scree plot suggested a one component solution. The second component had an eigenvalue of 1.48 and explained 12.35% of the variance.

**Table S3. Principal Component Analysis (PCA) of Altruism and Personal Benefits Items.**

| **Item** | **Component 1 loading** | **Component 2 loading** |
| --- | --- | --- |
| I support my partners participation in MAP to advance research about Alzheimer disease. | 0.09 | 0.90 |
| I support my partners participation in MAP to benefit society. | 0.05 | 0.90 |
| I support my partners participation in MAP to benefit future generations of my family. | 0.20 | 0.80 |
| I support my partners participation in MAP because I have concerns about their memory. | 0.67 | -0.05 |
| I support my partners participation in MAP to gain access to support at the medical center (advice and expertise). | 0.87 | 0.12 |
| I support my partners participation in MAP to learn more about Alzheimer disease and other dementias. | 0.68 | 0.41 |
| I support my partners participation in MAP to have access to future state-of-the-art treatments and information. | 0.78 | 0.25 |
| I support my partners participation in MAP because they enjoy spending time with the staff. | 0.63 | 0.06 |
| Eigenvalue of component | 3.50 | 1.75 |
| % variance explained | 43.73% | 21.85% |
| Cronbach’s alpha | 0.79 | 0.86 |

*Note*. Only two components were extracted that had an eigenvalues above 1.00; the next highest eigenvalue was 0.73. When calculating Cronbach’s alpha, the last 5 items were included in the Component 1 Cronbach’s calculation, while the first 3 items were included in the Component 2 Cronbach's calculation.

**Part 4: Comparison of participants who completed the survey online vs. mail**

|  | **Online via REDCap (*n* = 232)** | **Postal mail (*n* = 285)** |  |  |
| --- | --- | --- | --- | --- |
| **Component scores** | ***Mean (SD)*** | ***Mean (SD)*** | ***t*** | ***p*** |
| Perceived burden | -0.40 (0.94) | 0.03 (1.03) | 0.72 | 0.47 |
| Altruism | -0.06 (1.16) | 0.05 (0.84) | 1.21 | 0.23 |
| Personal benefits | 0.03 (1.04) | -0.02 (0.96) | -0.52 | 0.61 |
| Commitment | -0.01 (0.97) | 0.01 (1.03) | 0.21 | 0.84 |
| Remuneration | -0.06 (1.00) | 0.05 (1.00) | 1.18 | 0.24 |
