# Supplementary material for: Study partners’ views on remuneration in longitudinal Alzheimer’s disease research
Source: Alzheimers Dement Behav Socioecon Aging. Author manuscript; Available in PMC 2025 Dec 6. (PMC12680024; doi:10.1002/bsa3.70048)
Supplement: Support 2 Figures [file NIHMS2123218-supplement-Support_2_Figures.docx]

**Part 5: Figure S1. Parallel Test for Component Retention in Table 2: Observed, Adjusted, and Random Eigenvalues**
